# Transcriptome and methylome analysis reveals three cellular origins of pituitary tumors

**DOI:** 10.1038/s41598-020-76555-8

**Published:** 2020-11-09

**Authors:** Keiko Taniguchi-Ponciano, Sergio Andonegui-Elguera, Eduardo Peña-Martínez, Gloria Silva-Román, Sandra Vela-Patiño, Erick Gomez-Apo, Laura Chavez-Macias, Guadalupe Vargas-Ortega, Laura Espinosa-de-los-Monteros, Baldomero Gonzalez-Virla, Carolina Perez, Aldo Ferreira-Hermosillo, Etual Espinosa-Cardenas, Claudia Ramirez-Renteria, Ernesto Sosa, Blas Lopez-Felix, Gerardo Guinto, Daniel Marrero-Rodríguez, Moises Mercado

**Affiliations:** 1 CONACyT-Unidad de Investigación Medica en Enfermedades Endocrinas, Hospital de Especialidades, Centro Medico Nacional Siglo XXI, Instituto Mexicano del Seguro Social, Av. Cuauhtémoc 330, Col. Doctores, 06720 Mexico, D.F. Mexico; 2 Área de Neuropatología, Servicio de Anatomía Patológica, Hospital General de México Dr. Eduardo Liceaga, Mexico City, México; 3 Facultad de Medicina, Universidad Nacional Autonoma de México, Mexico City, México; 4 Servicio de Endocrinologia, Hospital de Especialidades, Centro Medico Nacional Siglo XXI, Instituto Mexicano del Seguro Social, Mexico City, Mexico; 5 Servicio de Neurocirugia, Hospital de Especialidades, Centro Medico Nacional Siglo XXI, Instituto Mexicano del Seguro Social, Mexico City, Mexico

**Keywords:** DNA methylation, Transcriptomics, Endocrine system and metabolic diseases, Molecular medicine, Diagnostic markers, Predictive markers, Pituitary tumours, Mechanisms of disease

## Abstract

Pituitary adenomas (PA) are the second most common intracranial tumors. These neoplasms are classified according to the hormone they produce. The majority of PA occur sporadically, and their molecular pathogenesis is incompletely understood. The present transcriptomic and methylomic analysis of PA revealed that they segregate into three molecular clusters according to the transcription factor driving their terminal differentiation. First cluster, driven by NR5A1, consists of clinically non-functioning PA (CNFPA), comprising gonadotrophinomas and null cell; the second cluster consists of clinically evident ACTH adenomas and silent corticotroph adenomas, driven by TBX19; and the third, POU1F1-driven TSH-, PRL- and GH-adenomas, segregated together. Genes such as *CACNA2D4, EPHA4 and SLIT1,* were upregulated in each of these three clusters, respectively. Pathway enrichment analysis revealed specific alterations of these clusters: calcium signaling pathway in CNFPA; renin-angiotensin system for ACTH-adenomas and fatty acid metabolism for the TSH-, PRL-, GH-cluster. Non-tumoral pituitary scRNAseq data confirmed that this clustering also occurs in normal cytodifferentiation. Deconvolution analysis identify potential mononuclear cell infiltrate in PA consists of dendritic, NK and mast cells. Our results are consistent with a divergent origin of PA, which segregate into three clusters that depend on the specific transcription factors driving late pituitary cytodifferentiation.

## Introduction

Pituitary adenomas (PA) are monoclonal epithelial tumors arising from adenohypophyseal cells, representing 10–15% of all intracranial tumors and occurring in almost 20% of the general population^1^. These tumors can be classified as clinically functioning and non-functioning PA. Clinically functioning PA are associated with specific hormonal hypersecretion syndromes such as acromegaly due to GH-secreting somatotrophinomas, galactorrhea/amenorrhea/sexual dysfunction due to PRL-secreting prolactinomas, Cushing disease due to ACTH-secreting corticotrophinomas and the rare TSH-secreting thyrotrophinomas causing secondary hyperthyroidism^2^. Clinically non-functioning PA (CNFPA) do not produce a distinct hormonal hypersecretion syndrome and usually present with compressive symptoms and signs such as headache and visual field defects. Most CNFPA are in fact neoplasms of gonadotroph differentiation since they immunostain for α-subunit, LHβ and/or FSHβ, as well for the transcription factor NR5A1 (also known as SF-1), followed by null cell PA which stain neither for any hormones, nor for any transcription factors and finally silent corticotroph, somatotroph or lactotroph PA^3^. Although considered benign, as many as 25–55% of pituitary PA have a clinically aggressive behavior and invade nearby structures^1,4^.

The majority of PA are sporadic with less than 5% occurring in a familial or hereditary context in which germline mutations of specific genes account for their oncogenesis (Multiple Endocrine Neoplasia type 1 and 4 [MEN1 AND 4], Familial isolated Pituitary Adenoma [FIPA], Carney Complex)^5^. Genomic characterization of sporadic PA has revealed *USP8* gene mutations in over 40% of ACTH-producing adenomas^6^ and *GNAS* mutations in 40% of GH-producing adenomas^7^. No other single gene or hot spot mutations have been found in sporadic PA^8^. In recent years genomic efforts to elucidate the molecular etiology of PA has revealed that mutational burden and copy number variation (CNV) are uncommon^7,9^ and thus, other molecular mechanisms are likely to be involved in PA tumorigenesis^10^. Furthermore, elucidation of the molecular basis of these tumors may expand therapeutic strategies.

In the present work we performed a detailed transcriptome and methylome analysis and a comprehensive bioinformatic analysis in one of the most complete series of PA of different types. Our objectives were to elucidate the potential cell of origin of these neoplasms and to identify the cellular pathways involved in their tumorigenesis in order to better guide future therapeutic pathways.

## Results

### Clinical characteristics and immunohistochemical phenotypification of PA

We evaluated a total of 6 non-tumoral pituitaries and 42 PA: 20 (47.6%) were non-functioning adenomas (14 gonadotrophinomas, 3 null cell adenomas and 3 silent ACTH-secreting adenomas); 10 (24%) were GH-secreting adenomas; 2 (4.7%) were prolactinomas, 4 (9.5%) were TSH-secreting adenomas and 6 (14.2%) were clinically manifest ACTH-secreting adenomas (Cushing disease).

### PA transcriptome reflects three different cell origins

**Figure 1:**
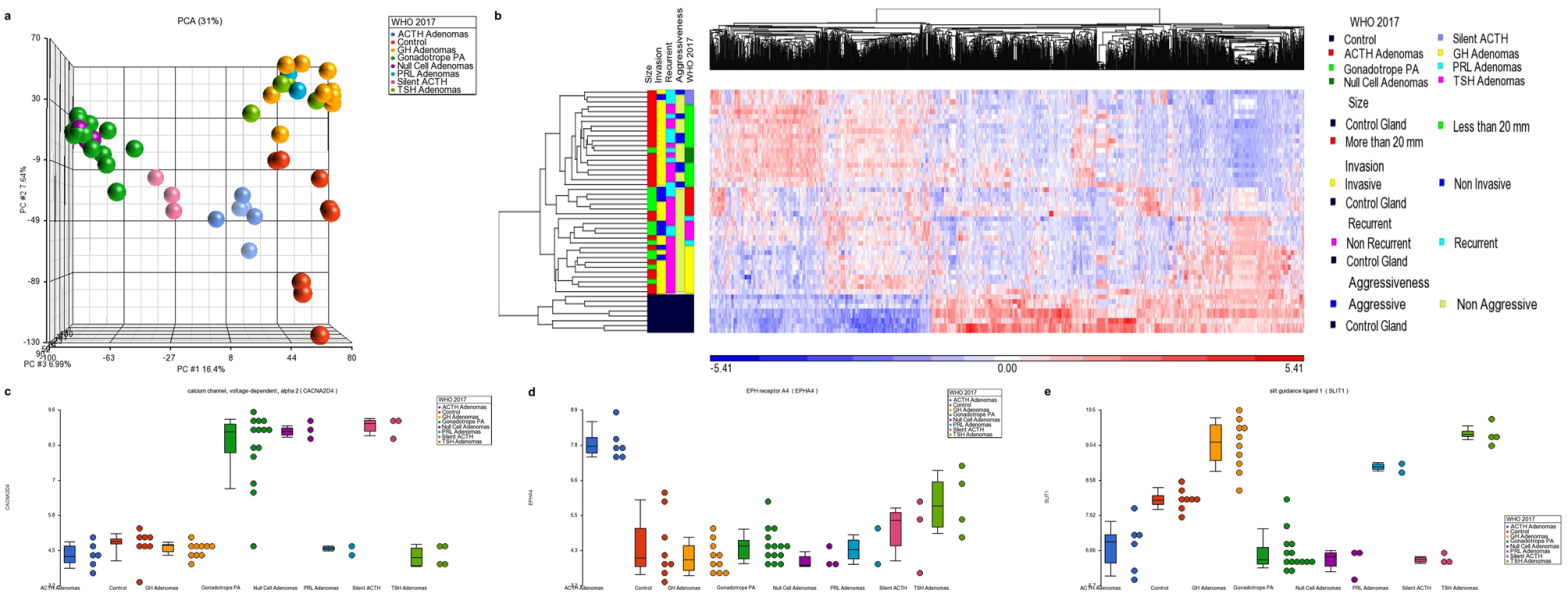
(**A**) PCA of pituitary adenoma (PA) transcriptome showing three distinct clusters: POU1F1-driven GH-, TSH- and PRL-adenomas; NR5A1-driven gonadotropinomas and null cell adenomas; and TBX19-driven clinically evident ACTH adenomas. Silent ACTH PA grouped separately sharing features with both, TBX19-dependent and NR5A1-dependent adenomas. (**B**) Heatmap of the differentially expressed genes. In the “Y” axis tumor samples are grouped according to the World Health Organization (WHO) 2017 classification as gonadotrope cell adenomas, null-cell adenomas, clinically evident ACTH adenomas, clinically silent ACTH adenomas, somatotrope adenomas, prolactinomas and TSH adenomas; tumors are also classified in the figure according to clinical features such as size, invasion, recurrence and aggressiveness. The “X” axis represents the differentially expressed genes hierarchical cluster. (**C**) CACNA2D4 is upregulated in NR5A1-driven tumors; (**D**) EPHA4 is upregulated in TBX19-driven tumors; (**E**) SLIT1 is upregulated in POU1F1-driven tumors. Image was created using Partek Genomics Suite 7.19v (https://www.partek.com/partek-genomics-suite/).

Principal component analysis (PCA) revealed the presence of three distinct transcriptomic clusters. The first cluster encompassed all CNFPA of gonadotroph differentiation and including the null cell adenomas. In the second cluster we found all the ACTH-producing adenomas causing Cushing disease. Silent ACTH adenomas segregated separately in a transcriptomic cluster that shared features with both, the CNFPA and the clinically manifest ACTH adenomas. The third cluster consisted of all the clinically manifest GH, PRL and TSH adenomas. Thus, transcriptomically, PA appeared to segregate and cluster according to the driving transcription factor responsible for terminal cytodifferentation of the pituitary gland: NR5A1 in the case of gonadotrophinomas, null cell and silent adenomas; TBX19 in the case of clinically manifest ACTH-adenomas; and POU1F1 in clinically manifest GH, PRL and TSH adenomas, indicating potentially, three divergent origin of the PA (Fig. 1).

Common up-regulated genes among each one of the PA subtypes were observed, potentially representing the collective tissue origin from which they derive. Genes such as *SLC25A2*, *LINC00412* and miR590 were up regulated in all PA. The clinically functioning ACTH-adenomas cluster was characterized by up-regulation of genes *AVPR1B*, *CRHR1* and *EPHA4*. The CNFPA clustered together showing minor differences in their transcriptome regardless as to whether they were gonadotrophinomas, null cell-, or silent ACTH adenomas. Remarkably, silent ACTH adenomas, gonadotrophinomas and null cell adenomas clustered together showing up-regulation of genes such as *CACNA2D4* and *EPHB6*. The cluster comprising GH-, PRL- and TSH functioning PA was characterized by upregulation of genes such as *SLIT1*, *PRLR* and *SLC16A6* (Fig. 1) (Supplementary Figure 1). PRL- and TSH-secreting tumors, but not GH-tumors, tended to further share differentially expressed genes such as *ADGRF2* and *FAM122A*.

Our transcriptomic analysis included the identification of non-coding RNA’s such as micro-RNA (miRNA), long non-coding (lncRNA) and circular RNA (cRNA). The expression pattern of these RNA species segregated into the three different tumor clusters. The ACTH-adenoma cluster overexpressed miR4501, the CNFPA cluster overexpressed miR582, miR4774 and *LINC01351*, while the GH- TSH- and PRL-adenoma cluster overexpressed miR377 and miR136 (Supplementary Figure 2).

### Cell clusters identified in tumors validated by single cell RNAseq in non-tumoral pituitary

**Figure 2:**
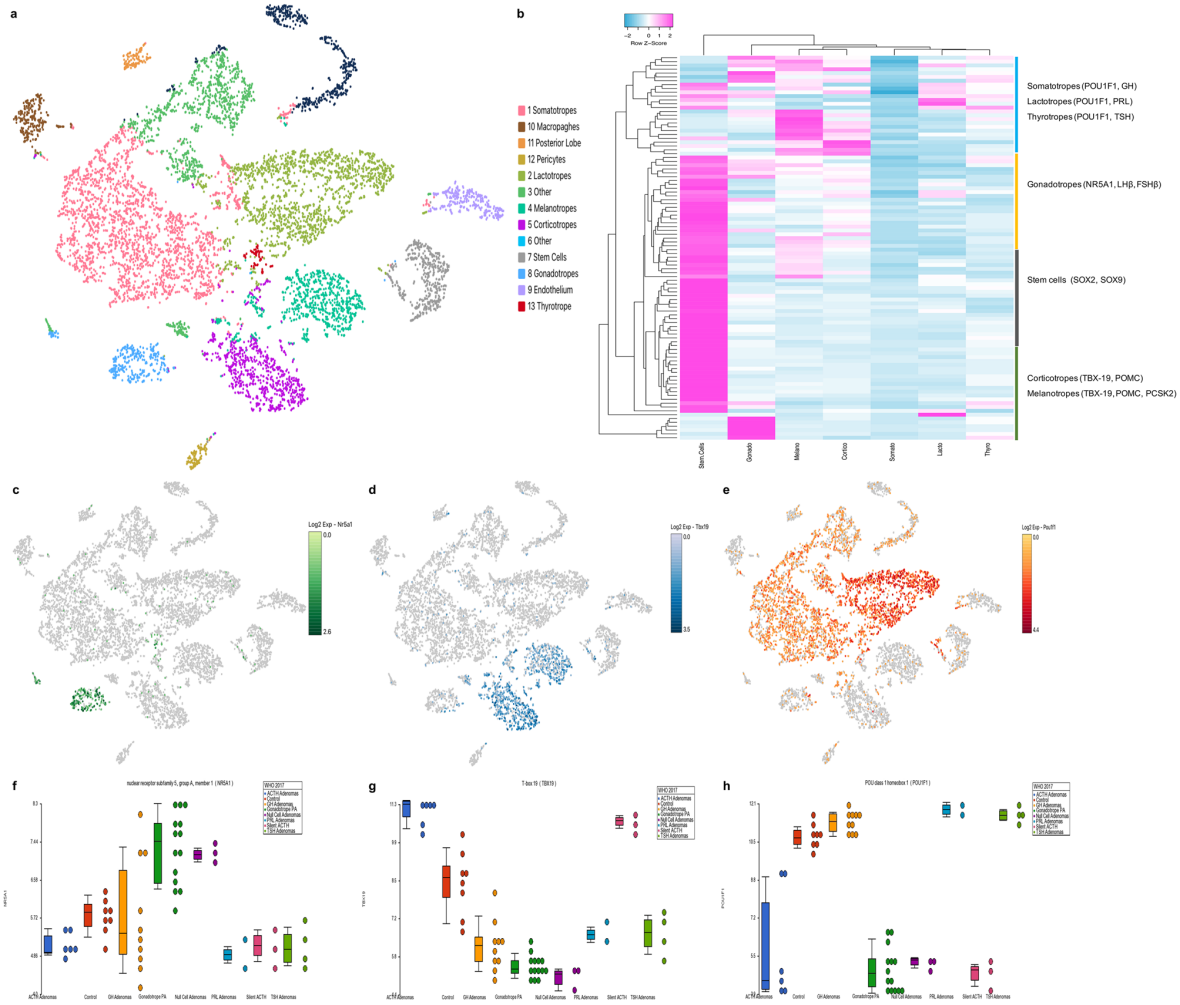
(**A**) t-distributed stochastic neighbor embedding map (t-SNE) showing the identification of 13 cell clusters using unsupervised k-means clustering. Clusters were identified using hallmark gene expression for each cell type. (**B**) The heatmap depicts the differentially expressed genes (Y axis), among the different pituitary cell types (X axis). Stem cells have a distinct transcriptome, whereas the different pituitary cells cluster according to the transcription factor responsible for their terminal differentiation: the gonadotrope cluster (NR5A1-driven), the corticotrope and melanotrope cluster (TBX19-driven) and the somatotrope-lactotrope-thyrotrope cluster (POU1F1-driven). (**C**–**E**) depict the distinctive pituitary cell populations expressing each of these transcription factors. (**F**–**H**) show the expression of each of these transcription factors by the different types of pituitary adenomas: NRA5A1 highest expression occurs in CNFPA (gonadotropinomas and null cell), TBX19 highest expression is found in adrenocorticotropinomas and POU1F1 highest expression was observed in somatotrope, lactotrope and thyrotrope adenomas. Image was created using Partek Genomics Suite 7.19v (https://www.partek.com/partek-genomics-suite/) and Loupe Cell Browser (https://www.10xgenomics.com).

We performed a metanalysis of publicly available mouse non-tumoral pituitary single cell RNAseq data to validate our findings. Interestingly, transcriptomic analysis from non-tumoral pituitaries coincided with our findings in tumoral tissues: gonadotrophs have high transcriptional levels of NR5A1; corticotrophs overexpress TBX19; and lactotrophs, somatotrophs and thyrotrophs cluster together as they overexpress POU1F1 (Fig. 2). A significant correlation was found between our trancriptomic data and the scRNAseq results regarding the mRNA expression of canonical cell lineage molecular markers such as TSHβ, FSHβ, GH, POMC and PRL (Supplementary Figure 3). These results showed that tumors originated from at least three divergent progenitor cells which correlate with the three transcription factors that drive normal pituitary embryogenesis.

### Methylation patterns point to different cell origins and distinguishes tumor subtypes

**Figure 3:**
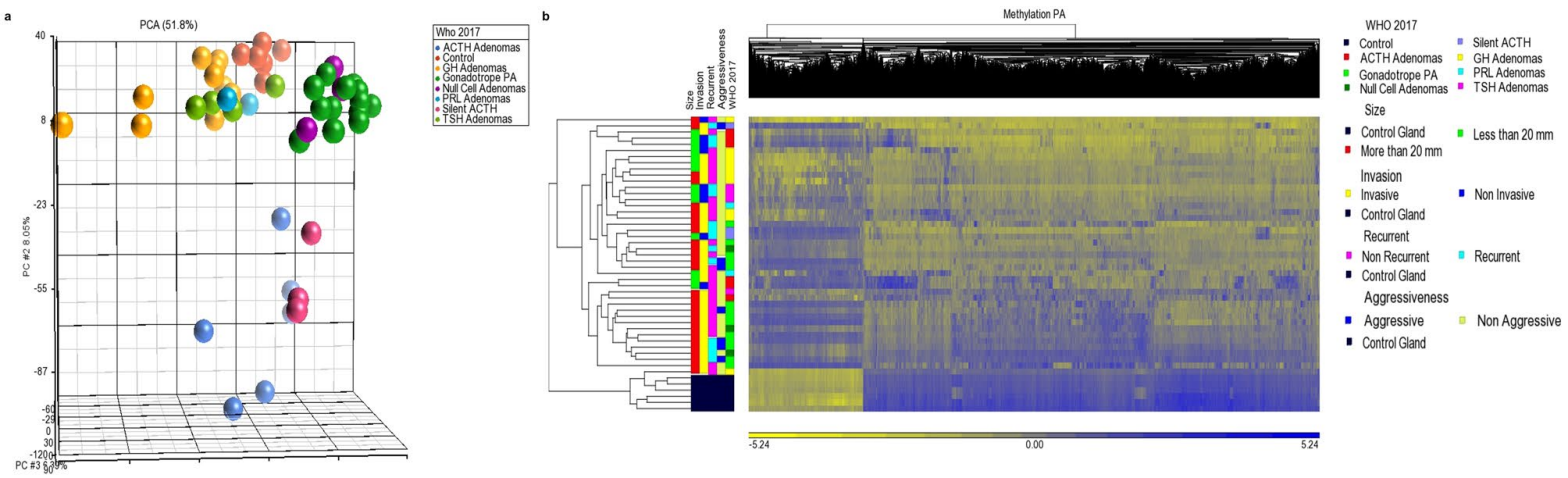
(**A**) Methylome PCA of the three pituitary adenoma clusters: POU1F1-driven somatotropinoma-thyrotropinoma-prolactinoma cluster; NR5A1-driven gonadotropinoma and null cell adenoma cluster; and TBX19-driven adrenocorticotropinoma cluster. Silent ACTH-adenomas segregated separately between TBX19-adenomas and NR5A1-adenomas. (**B**) Differentially methylated genes heatmap clustering POU1F1-derived adenomas, NR5A1-derived adenomas and TBX19-adenomas which split in two groups, one cluster next to POU1F1 adenomas and the second next to NR5A1 adenomas. The “Y” axis represents the tumor sample clustering according to WHO 2017 classification, as well as clinical features such as size, invasion, recurrence and aggressiveness, while the “X” axis represents the methylated regions hierarchical cluster. Image was created using Partek Genomics Suite 7.19v (https://www.partek.com/partek-genomics-suite/).

Overall, a hypomethylated state was observed in PA when compared to non-tumoral gland. PCA methylome analysis identified the same three clusters described in the transcriptomic analysis: (1) non-functioning adenomas grouped together irrespective of their immunophenotype; (2) GH-, PRL- and TSH-secreting adenomas shared the same methylome pattern; and (3) ACTH-secreting adenomas clustered together (Fig. 3). Again, silent ACTH adenomas segregated separately in a cluster that shared features with both the CNFPA and the clinically manifest ACTH adenomas. The differentially methylated genes again showed the three groups, ACTH adenomas divided in two distinct gene-silencing patterns, one adjacent to non-functioning tumors cluster and the other adjacent to the GH-, TSH- and PRL cluster (Fig. 3). In order to identify potentially methylation-regulated genes, the transcriptomic and methylomic information was analyzed together.

In non-functioning adenomas, the expression of approximately 1066 genes was found to be potentially controlled by methylation. The expression of genes such as *CACNA2D4, GRIA2*, miR4774, miR1179 and LINC01351 was found to be up-regulated due to lower methylation rates in non-functioning adenomas. In ACTH-secreting tumors approximately 649 genes were found to be potentially regulated by DNA methylation, including genes such as *AVPR1B, EPHA4, GRIN2B, GRIA4*, miR592 and miR4796 that were upregulated by promoter demethylation. In GH-, PRL- and TSH-secreting adenomas genes such as *SLIT1*, *PRLR* and *miR377* were upregulated due to demethylation. More specifically, 204 genes in PRL adenomas and 184 genes in GH adenomas, were found to have an altered expression due to a distinct methylation pattern, including *GRIN3A* in the former and *TMEM233* and *GRIA4* in the later. Finally, TSH adenomas showed 68 genes that could be regulated by methylation including *SSTR2*, *GRIA2* and LINC01173.

### Potential molecular therapy targets in PA

We identified several up-regulated genes in the different types of PA that could eventually become specific targets for molecular therapy. Druggable genes shared by all types of pituitary adenomas included *NRG1, KCNA4, NCAM1, GRIA2 and GRM8*. More specifically, genes with such a therapeutic potential in non-functioning adenomas included *CACNA2D4, IDH1, AURKB, GRIA2, PTGS2 and CX3CR1*. Other tumor-type-specific “druggable” genes included *EPHA4, AVPR1B, CACNA2D2, AR, CCL3 and PTGER4* in ACTH-secreting adenomas; *SLIT1, BRCA2, DYRK1B, GHSR, and GRM5* in GH-secreting adenomas; *SLIT1, KCNH8, GRM5, GRIN3A and NRG1* in prolactinomas; *SLIT1, SSTR2*, *KCNA4, KCNQ5, GRIA1, and GRIA3* in TSH-secreting adenomas; and *SLIT1, KCNH8, GRM5, GRIN3A, NRG1* in prolactinomas.

### Gene validation

**Figure 4:**
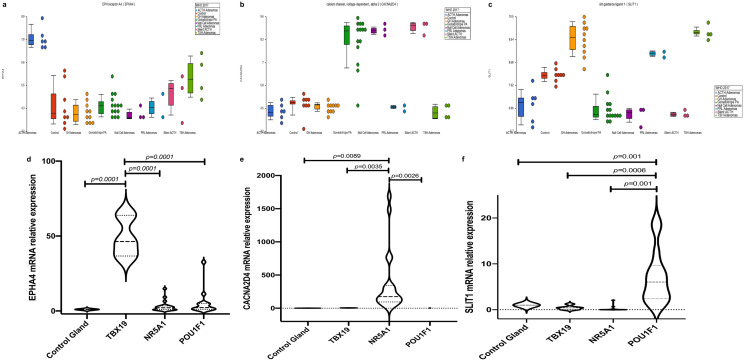
(**A**–**C**) Dot blots of representative up regulated genes. *EPHA4* in TBX19-derived tumors*, CACNA2D4* in NR5A1-derived tumors and *SLIT1* in POU1F1-derived tumors. (**D**–**F**) Violin plots showing validation of gen up-regulation in each tumor group by RT-qPCR. Image was created using Partek Genomics Suite 7.19v (https://www.partek.com/partek-genomics-suite/).

Representative genes for each observed cluster were selected on the basis that they are up-regulated as archetypal of their respective tumor subtype, that they showed hypomethylation status and that they represent potentially therapy targets in our drug-gene interaction analysis. Compared to non-tumoral pituitary, *EPHA4* was 51.5, 5.25, and 3.03 times more up-regulated in ACTH-secreting tumors, TSH, PRL and GH-secreting adenomas and non-functioning adenomas, respectively (p = 0.0001). *SLIT1* was particularly more up-regulated in TSH-, GH- and PRL-secreting adenomas (7.16) than in ACTH-adenomas (0.425) or non-functioning adenomas (0.172), (p = 0.0001). *CACNA2D4* expression was significantly more up-regulated in non-functioning adenomas (367.03) than in ACTH-secreting adenomas (1.659) or TSH-, GH- and PRL-secreting tumors (0.797) (p = 0.0089) (Fig. 4).

### Pathway enrichment analysis

**Figure 5:**
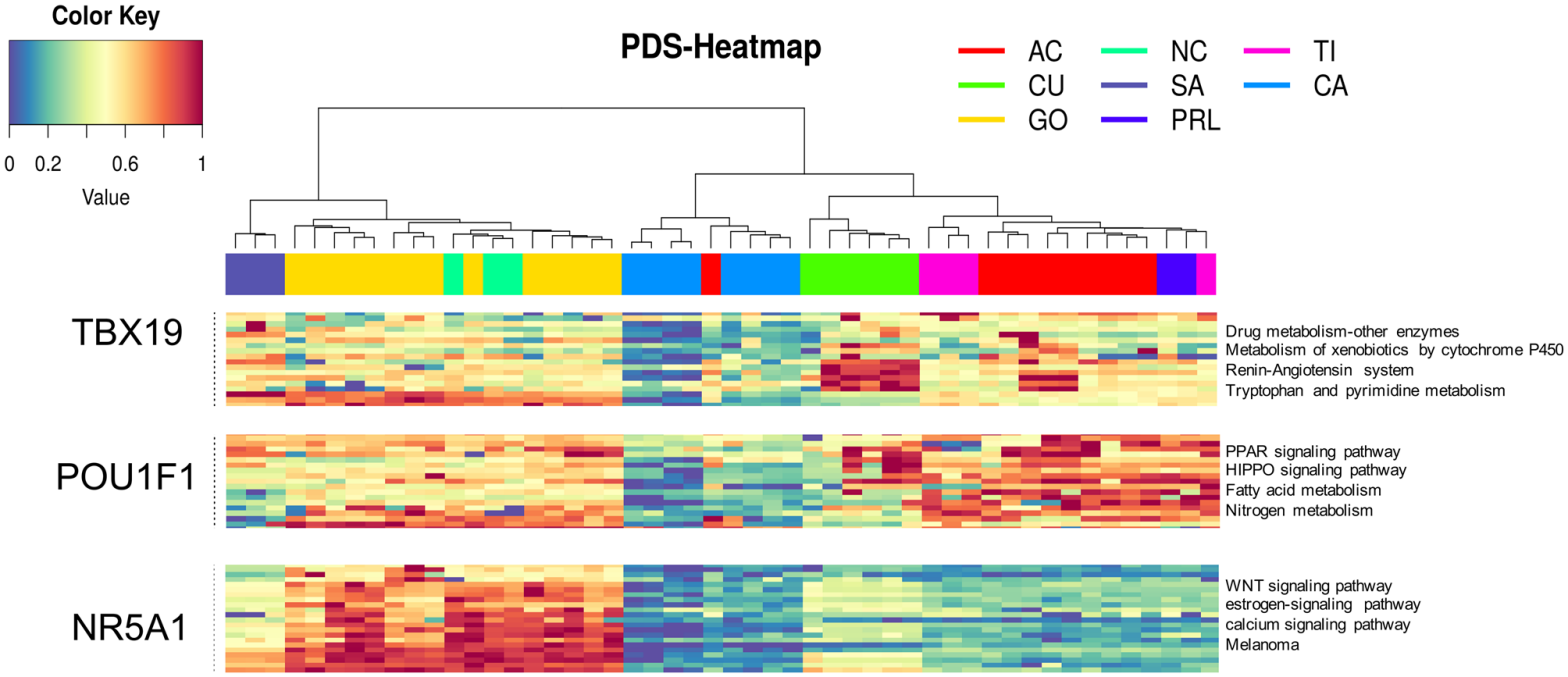
Representative deregulated pathways in the TBX19-, POU1F1- and NR5A1-derived tumors. Altered cellular events cluster the tumors into the respective transcription factors they derive from. Silent ACTH tumors resemble the NR5A1-driven cells more than the TBX19-driven cells and therefore share molecular events common to both cell lineages. CA = control, non-tumoral gland, which has a PDS of 0; AC = GH tumors causing acromegaly; NC = clinically non-functioning null cell adenomas; CU = ACTH tumors causing Cushing’s disease; GO = gonadotropin tumors causing clinically non-functioning adenomas; SA = silent ACTH, clinically non-functioning tumors; PRL = prolactin-secreting prolactinomas; TI = TSH-secreting tumors or thyrotropinomas.

Several of the genes found to be differentially expressed in this transcriptomic study encode a myriad of proteins that are key participants in many signaling pathways. In CNFPA we found differentially expressed genes which encoded proteins are involved in WNT signaling pathway, estrogen-signaling pathway, calcium signaling pathway and immune related events such as Th17 cell differentiation. Pathways involved in ACTH-secreting tumors included drug metabolism enzymes, metabolism of xenobiotics by cytochrome P450, renin-angiotensin system, tryptophan and pyrimidine metabolism and those involved in immune related events such as in systemic lupus erythematosus. The transcriptome of POU1F1-dependent GH, PRL and TSH-secreting tumors revealed differentially expressed genes whose encoded proteins participate in fatty acid metabolism and nitrogen metabolism, as well as PPAR and HIPPO signaling pathways (Fig. 5). Genes involved in cellular senescence were up-regulated predominantly in GH-, PRL- and TSH-secreting tumors, as well as in CNFPA compared to ACTH-secreting lesions, whereas in all tumors, genes involved in calcium, spliceosome and fatty acid metabolism as well as in immune-related events were found to be up regulated. Such immune-related events include, among others, antigen processing and presentation, Natural killer cell-mediated toxicity, TGFβ and TNF signaling pathways, Th17 cell differentiation, chemokine signaling pathway, cytokine-cytokine receptor interaction and the IL-17 signaling pathway.

### Immune cell infiltrates in pituitary PA

**Figure 6:**
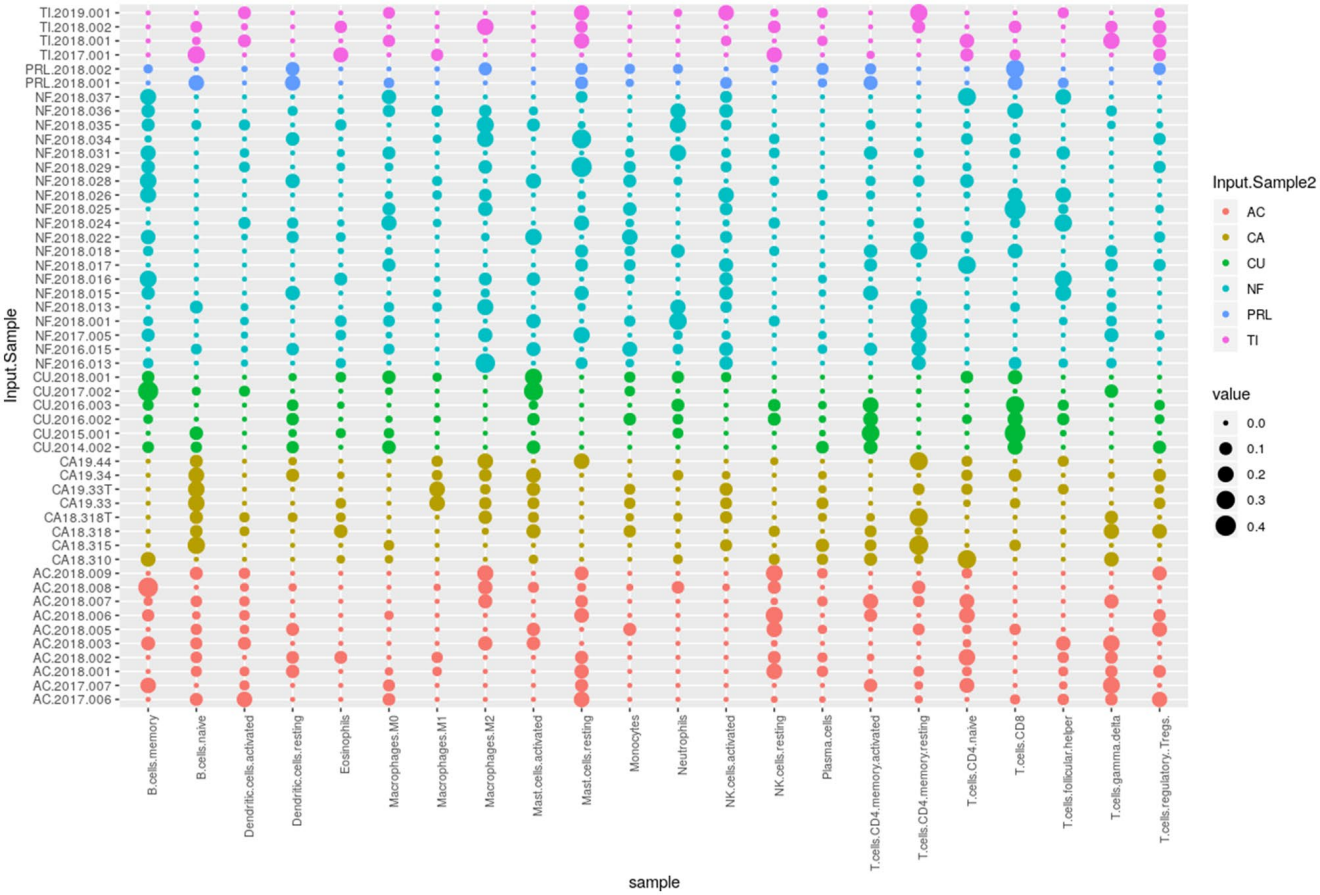
Dot blot plots of the deconvolution analysis showing the type of immune cell infiltration found in each tumor. Dendritic cells, CD4 + and CD8 + T lymphocytes as well as macrophages were among the most commonly found infiltrating immune cells. CA = control, non-tumoral gland; AC = GH tumors causing acromegaly; NC = clinically non-functioning null cell adenomas; CU = ACTH tumors causing Cushing’s disease; GO = gonadotropin tumors causing clinically non-functioning adenomas; SA = silent ACTH, clinically non-functioning tumors; PRL = prolactin-secreting prolactinomas; TI = TSH-secreting tumors or thyrotropinomas.

After the identification of immune-related events in the pathway analysis we carried out cellular deconvolution analysis throughout the whole transcriptome to identify which immune system cells that may be infiltrating PA. Cellular deconvolution analysis through the whole transcriptome revealed different immune system cells that could be infiltrating PA in varying degrees. Interestingly, the deconvolution analysis revealed the presence of several types of immune cells infiltrating the PA. According to this analysis the infiltrating immune cells were natural killer and mast cells, but other cell types, such as CD4+ and CD8+ T lymphocytes, as well as macrophages were also present (Fig. 6). Reviewing the H&E stains of several PA we found that some of them had a clear mononuclear cell infiltrate, which supports our molecular observations found through deconvolutional analysis (Supplementary figure 4). The interleukin expression profile showed the formation of a very similar pattern of the three previously observed groups (Supplementary figure 5), whereas the chemokines profile does not seem to differentiate into these three clusters.

## Discussion

The anterior lobe of the pituitary gland consists of several highly specialized cell populations that synthesize and secrete specific hormones. The diverse cell types and their corresponding hormone expression profiles have been traditionally used to classify PA. While the multicellular milieu and hormonal subtypes of these tumors contribute to intra- and intertumoral heterogeneity, the tumorigenesis of PA remains incompletely understood^11^. Tumor stem cells (TSC) are a pool of relatively undifferentiated cells that have the capacity for self-renewal and that can differentiate into any of the cells that constitute a tumor^12,13^. The anterior pituitary contains populations of stem cells, that are the progenitors of the different hormone-producing cells during development and postnatal life^14^. Stem-like cells have also been identified in PA raising the possibility that they represent a tumor-initiating cell population^13,15–17^. However, a pituitary TSC phenotype has not been completely defined^18^.

Our transcriptomic and methylomic results, as well as our pathway analysis findings support the notion that there at least three different TSC populations from which three distinct types of PA develop, based on their canonical transcription factors, namely, TBX19-driven corticotrophiomas, NR5A1-dependent CNFPA (gonadotrophinomas and null cell adenomas) and POU1F1-dependent GH-, PRL and TSH adenomas^3^. These results coincide with our in-silico analysis of non-tumoral pituitary scRNAseq data. Interestingly, there is a significant intertumoral heterogeneity indicating a spectrum of stem cell populations within the different PA subtypes^11^. Nevertheless, virtually all CNFPA clustered together as one group, regardless of their immunophenotype as gonadotroph or null-cell. This clustering of CNFPA was previously reported by our group^19^ and correlates with the clinical behavior of these tumors^20^. Similarly, the clustering of POU1F1-dependent tumors is congruent with the finding of plurihormonal tumors capable of synthesizing GH, PRL and TSH in different combination^21^. Thus, it appears that these three transcription factors drive not only normal gland cytodifferentiation but also pituitary tumorigenesis. These results also suggest that PA progenitor cells do not originate early in the cytodifferentiation process but derive instead from an already partially committed cell. Undoubtedly, further elucidation of the mechanisms underlying pituitary stem cell self-renewal, differentiation and programmed death may lead to a better understanding of pituitary homeostasis, plasticity and tumorigenesis^15^.

Pathway enrichment analysis of our transcriptomic results revealed potential molecular targets for the design and development of novel therapies. We found a large number of altered genes involved in the regulation of the biological actions of calcium, glutamate and potassium. Calcium, glutamate and potassium ions play a central role in biological systems; they can induce and transduce intracellular signaling by binding to a plethora of proteins. Ion channels are pore-forming transmembrane proteins that regulate passive ion fluxes that are crucial for several cellular processes and are known to be expressed in different human tumors^22^. These processes include cellular proliferation, differentiation, invasion and apoptosis control, nitrogen and intermediate metabolism, macromolecule synthesis as well as epigenetic events^23–27^. Besides being potential tumor molecular markers, they could represent targets for novel molecular therapies. There are currently several drugs targeting ion channels/transporters such as ATPase inhibitors^25,28,29^ that have been shown to effectively reduce tumor size^30^.

The study of epigenetics has opened a new perspective in the diagnosis, treatment and follow up of human disease. The identification of methylation-regulated genes in specific tumors is currently being used with diagnostic, prognostic and therapeutic purposes^31,32^. There are currently several epigenetic therapies, which in combination with traditional therapies have shown significant improvements in response^33^. In the present study we found a large number of methylation-regulated genes that segregated with the three main tumor types and that could be used as targets for new molecular therapies.

A non-negligible proportion of PA has been found to have mononuclear cellular infiltrates. Immune system cells potentially infiltrating the tumor mass could influence tumor microenvironment and finally tumor behavior. The infiltration of NK cells could potentially improve antitumor immune responses^34^. NK cells represent a host-dependent hallmark and key paradigm in tumor progression and thus, could be suitable targets for immune therapy^35^. Mast cells have been involved in both, tumor promotion and suppression; their role in tumor biology is still unclear and appears to be dependent on their microlocalization as well as on tumor subtype^36^. The use of certain mast cells modulators could improve the efficacy of anti-tumor therapy in certain cases^37^. Macrophage infiltration could potentially be involved in epithelial-mesenchymal transition and could render a tumor to behave more aggressivel^38^. The presence of M2 polarized macrophage cells but also the presence of T Cell species has been related to tumor size and invasiveness in PA^39^. Immune cell infiltration in PA has proven to be an indicator of poor clinical outcome^40^. Deconvolutional analysis of our transcriptomic data showed that immune cells such as NK cells, mast cells as well as CD4 and CD8 lymphocytes could be present in various degrees within PA. A better understanding of the immune microenvironment of PA may bring us closer to the development of safe and effective immune treatments, for example, targeting tumor infiltrating cells to constrain tumor growth and invasiveness^38,41^.

In conclusion, our results are consistent with a divergent origin of PA, which segregates transcriptomically into three distinct clusters that depend on the specific transcription factors that drive late pituitary cytodifferentiation. Our data can potentially be used to tailor novel and effective molecular therapies that could halt the progression of these neoplasms.

## Materials and methods

### Patients and tissue samples

Forty-two tissue samples were collected, including: twenty CNF (14 gonadotrope, 3 null cell and 3 silent ACTH) PA, ten GH PA, six ACTH PA, four TSH PA and two PRL PA. All tumors were collected from patients diagnosed treated and follow at the Endocrinology Service and the Neurosurgical department of Hospital de Especialidades, Centro Médico Nacional Siglo XXI of the Instituto Mexicano del Seguro Social from May 2016 to May 2019. All tissue samples were from treatment naïve patients who had not received radiation therapy or any other pharmacological intervention prior to surgery, when possible. Six non-tumoral pituitary glands were obtained within 10 h of death from autopsies performed at the Pathology Department of Hospital General de México and were used as controls. All participating patients were recruited with signed informed consent and ethical approval from the Comisión Nacional de Ética e Investigación Científica del Instituto Mexicano del Seguro Social in accordance with the Helsinki declaration.

### RNA purification

Total RNA was extracted from PA and non-tumoral pituitaries using the miRNAeasy Mini Kit (Qiagen Inc, CA, USA) according to manufacturer’s instructions. Tissue samples were disrupted and homogenized in 700 μl Qiazol Lysis Reagent. They were then incubated at room temperature for 5 min. Next, 200 μl of chloroform was added, and samples were incubated at room temperature for 3 min. The mixture was centrifuged at 12,500 rpm for 15 min at 4 °C. The aqueous phase was transferred to a fresh tube and mixed with an equal volume of 70% ethanol. Samples were then transferred to an RNAeasy Column in a 2 ml tube, and centrifuged at 10,000 rpm for 15 s. After centrifugation, 700 μl of RW1 buffer was added and the mixture was centrifuged at 10,000 rpm for 15 s. Flow-through was discarded and 500 μl of RPE buffer was added to the membrane and then centrifuged at 10,000 rpm for 15 s (2×). The column was transferred to a new collection tube adding 30 μl of RNAse free water and centrifuged for 1 min at 10,000 rpm. RNA was quantified using a Nanodrop-ND-1000 spectrophotometer (Thermo Scientific, DE, USA); RNA integrity was evaluated by Bioanalyzer 2100^42^.

### DNA purification

Pituitary tissue was lysed in proteinase K solution. After lysis, 300 μl of 5 M ammonium acetate was added to precipitate proteins and cellular components. The aqueous phase was transferred to a fresh tube and 600 μl of isopropanol was added and incubating the mixture overnight at − 20 °C. The mixture was then centrifuged at 14,000 rpm for 30 min. The resulting DNA pellet was washed with 1 ml 75% ethanol and centrifuged at 10,000 rpm for 5 min; the pellet was air-dried, and DNA resuspended in nuclease free water.

### Reverse transcription and qPCR

After purification, 1 μg of total RNA was retro transcribed in a 20 μl final volume reaction with the SuperScript VILO Master Mix (Applied Biosystems, CA, USA), 4 μl of Master Mix were added, and the reaction mixture was incubated at 25 °C for 10 min., 42 °C for 60 min., and 85 °C for 5 min., according to manufacturer protocols. For RT-qPCR of SLIT1 (Hs00171488_m1), EPHA4 (Hs00953178_m1) and CACNA2D4 (Hs00297782_m1) all reagents were purchased from Applied Biosystems (CA, USA), and conditions were as follows: 10 μl of Taqman Universal Master Mix II, 1 μl of each Taqman probe, 200 ng of cDNA in a 20 μl final volume, according to manufacturer’s recommendation. RPLP0 (Hs99999902_m1) was used as endogenous control and all reactions were done in triplicate in the Step one thermal cycler (Applied Biosystems). 2^−ΔΔCt^ relative expression was calculated^42^.

### Microarray GeneChip Clariom D assay

The microarray used for these studies was Affymetrix Clariom D which allows us to analyze whole coding transcriptome at the gene and exon level as well as non-coding RNA such as lincRNA, miRNA and circRNA. Sample amplification and preparation for microarray hybridization was performed according to Affymetrix specifications. Briefly, 100 ng of total RNA was reversely transcribed into cDNA, amplified by in vitro transcription and reversely transcribed to cDNA again. Fragments between 40 and 70 bp were generated enzymatically, labelled and hybridized onto the microarray chips in an Affymetrix hybridization oven at 60 rpms and 45 °C for 17 h. Chips were washed according to the stablished protocols (Affymetrix, Santa Clara, CA, USA) with a GeneChip fluidics station 450, and finally scanned with an Affymetrix 7G GeneChip scanner. The raw data (CEL files) has been uploaded into the Gene Expression Omnibus (GEO), which is hosted by the National Center for Biotechnology Information (NCBI) under the accession number GSE147786.

### Bioinformatic analysis of PA transcriptome

A total of 6 control and 42 PA experiments were analyzed, and two technical replicates. Data sets were analyzed by means of CEL files with the Expression Console, Partek Genomics Suite 7.19v software (Partek Incorporated, Saint Louis, MO, USA) and the Transcriptome Analysis Console (Affymetrix, Santa Clara, CA, USA). Pearson and Spearman correlations were performed and probe sets were summarized by means of Median Polish and normalized by quantiles with no probe sets excluded from the analysis. Background noise correction was achieved by means of Robust Multi-chip Average (RMA) and data were log^2^ transformed. Data grouping and categorization was achieved by principal PCA. Differentially expressed genes were determined by means of ANOVA. Gene expression was considered to be altered upon identifying a + 2 or − 2-fold change compared to non-tumoral pituitaries, p ≤ 0.05 and FDR ≤ 0.05 parameters^19^.

### DNA methylation

The methylation profiles of PA were determined by means of the Infinium MethylationEPIC BeadChip Array (Illumina Inc.) following the manufacturer's protocol. Sodium bisulfite modification was performed on 1 μg of DNA using the Zymo EZ DNA methylation kit (Zymo Research). The treated DNA was whole-genome amplified and enzymatically fragmented. Finally, the amplified and fragmented DNA was hybridized to the MethylationEPIC BeadChip. The chips were scanned with the Illumina iScan.

### Bioinformatic analysis of the PA methylome

A total of 6 control and 42 PA experiments were analyzed. Data sets were analyzed by means of iDAT files with the Partek Genomics Suite 7.19v software (Partek Incorporated, Saint Louis, MO, USA). We exclude sexual chromosomes X and Y from our analyses, Functional normalization, which include NOOB background correction and dye correction, was carried out. Data grouping and categorization was achieved by principal component analysis (PCA). β-values obtained from the array was transformed to M-values using the formula M-value = log2 (β/(1 − β)), with the M-values the differentially methylated loci were determined by means of ANOVA. Methylated loci were considered altered with + 2- or – 2-fold change, p ≤ 0.05 and FDR ≤ 0.05 parameters.

### Pituitary single cell RNAseq analysis

The data corresponding to mouse single cell RNAseq was downloaded from publicly available data from the Gene Expression Omnibus website under accession number GSE125670. Data was analyzed and visualized using Loupe Cell Browser software from 10× Genomics to generate the transcriptome profile for each cell type in the pituitary gland.

### Deconvolution analysis

The deconvolution analysis was performed using the online tool CIBERSORT (Cell Type Identification By Estimating Relative Samples Of RNA Transcripts), which solves the equation m = f × B, where m is a mixture of mRNA, f is a vector that denotes the Cell fractions that make up the mixture and B is an array of gene expression profiles characteristic of each cell subtype. CIBERSORT is based on the application of v-SVR (nu-support vector regression) where limits of a hyperplane are determined that fits most of the possible points at a constant distance and where a regression is performed. Support vectors are genes selected from B. CIBERSORT was used for the present work with the default parameters, using the LM22 expression profile matrix (22 types of immune cells) and 100 permutations to calculate the p-value associated by Monte Carlo Sampling. The mRNA mixture used was a matrix constructed with the expression values ​​of each sample obtained by microarrays. The matrix and graphics were prepared using R version 3.6.0.

### Altered pathways identification

To analyze the path alteration, the PDS (pathway deregulation score) of each path noted in KEGG was calculated using the Pathifier algorithm. Pathifier calculates a PDS for each path for each sample. In each path a n-dimensional space is constructed (n = number of genes in the path), where a main curve that captures the variation of a cloud of points is calculated by non-linear regression, where each point represents each sample and its values of expression of the n genes of the pathway, PDS is the distance of the projection to the main curve of each sample with respect to the projection of normal samples. The analysis of this section was performed using R version 3.6.0. KEGG annotated tracks were downloaded using the “gage” package. An expression matrix was constructed using microarray expression values and PDS values were calculated using the "pathifier" package with the default parameters. Using the PDS values, samples and pathways were classified by the unsupervised hierarchical clustering method using Euclidean distance and the Ward method, available using the “dist” and “hclust” functions respectively. The heatmap was produced using the "heatmap.2" function.

### Drug-gene target interactions

Differentially expressed genes, particularly up-regulated genes were used to identify Drug-target associations through a tool known as The Drug-Gene Interaction Database (http://www.dgidb.org). FDA-approved, antineoplastic and immunotheraphy databases was used. This platform integrates information of at least 15 pharmacological databases which includes information about drugs, pharmacological targets, type of drug-target interaction, data sources, and other characteristics.

### Hormones and transcription factors immunohistochemistry

Paraffin-embedded, formalin-fixed tissue blocks were stained with hematoxylin–eosin and reviewed by a pathologist. Tumors were represented with a twofold redundancy, which has been shown to provide a sufficiently representative sample. Sections (3 μm) were cut and placed onto coated slides. Slides were deparaffinized with xylene followed by ethanol and rehydrated. Immunostaining was performed using HiDef detection HRP polymer system (Cell Marque, CA, USA).

Briefly, after dewaxing the sections, endogenous peroxidase activity was inhibited. Next, the sections were processed in a 600 W microwave oven at maximum power, three times for 5 min each in Tris–EDTA buffer (pH 9.0). Incubation with antibodies against TSH, GH, PRL, FSH, LH and ACTH hormones (M3501, M3502, M3504; A0569, A0570 Dako, CA, USA, CM412B BioCare Medical, CA, USA) and TBX19, POU1F1 and NR5A1 transcription factors (SC393592 Santa Cruz Biotechnology TX, USA, AB243028, Abcam, CA, UK, NBP1-92273, Novus Biologicals, CO, USA) was performed overnight at 4 °C in a humidity chamber at 1:100 dilutions, in 1% bovine serum albumin (BSA). Sections were developed with a peroxidase substrate solution, counterstained with hematoxylin, dehydrated, and mounted. Control pituitary was used as positive biological controls, and negative controls consisted of the replacement of the primary antibody with 1% BSA. Three independent observers performed assessment of hormones and transcription factors expression at different times^43^.

### Statistical analysis

Analysis was performed by means of the ANOVA with Tukey post-hoc for the RT-qPCR gene expression studies and for clinical characteristics. All p-values represent two-tailed tests and were considered significant when p < 0.05. Statistical software package consisted of v26 SPSS.

### Ethics approval and consent to participate

All participating patients were recruited with signed informed consent and ethical approval from the Comisión Nacional de Ética e Investigación Científica del Instituto Mexicano del Seguro Social in accordance with the Helsinki declaration. (R-2019-785-052).

## Supplementary information

Supplementary information 1.

Supplementary information 2.

Supplementary information 3.

Supplementary information 4.

Supplementary information 5.

Supplementary information 6.

## Data Availability

The raw data (CEL files) has been uploaded into the Gene Expression Omnibus (GEO), which is hosted by the National Center for Biotechnology Information (NCBI) under the accession number GSE147786. The data corresponding to single cell RNAseq was downloaded from the Gene Expression Omnibus web-site under accession number GSE125670.
