# Supplementary material for: Transcriptome and methylome analysis reveals three cellular origins of pituitary tumors
Source: Sci Rep. 2020 Nov 9;10:19373. doi: 10.1038/s41598-020-76555-8 (PMC7652879; doi:10.1038/s41598-020-76555-8)
Supplement: Supplementary file 1 — Supplementary information 1. [file 41598_2020_76555_MOESM1_ESM.docx]

**Figure S1.-** Panels A), B) and C) depict the up-regulation of SLC5A2, LINC00412 and miRNA590, respectively, in all pituitary tumors compared to control gland. D) and E) show AVPR1B and CRHR1 up-regulation in TBX-19-derived tumors. F) and G) portrays EPHB6 and ENO2 up-regulation in NR5A1-derived tumors. H) and I) denotes PRLR and SLC16A6 gene up-regulation in POU1F1-derived tumors and J) and K) depicts ADGRF2 and FAM122A gene up-regulation in PRL- and TSH- pituitary tumors. Image was created using Partek Genomics Suite 7.19v (https://www.partek.com/partek-genomics-suite/).

**Figure S2.-** Non-coding RNA segregates and categorizes each pituitary tumor cell lineage. A) miRNA, B) lincRNA and C) circRNA. Panels D), E), F), G), H) and I) portray up-regulation of non-coding genes miRNA4501, miRNA582, miRNA4774, lincRNA01351, miRNA377 and miRNA136, respectively, in the different tumor subtypes. Image was created using Partek Genomics Suite 7.19v (https://www.partek.com/partek-genomics-suite/).

**Figure S3.-** scRNAseq data showing canonical markers for each pituitary cell population: A) GH, B) TSH, C) PRL, D) FSHß, E) POMC, and corresponding to hormone expression in pituitary adenomas (F, G, H, I and J). Image was created using Partek Genomics Suite 7.19v (https://www.partek.com/partek-genomics-suite/) and Loupe Cell Browser (https://www.10xgenomics.com).

**Figure S4.-** Mononuclear cell infiltrates in pituitary adenomas. HE, A) and C) 40x, B) and D) 400x

**Figure S5.-** A). Interleukin gene expression categorizing tumor subtypes according to cell lineage. B). Chemokine gene expression profile shows a heterogeneous pattern across all tumor subtypes. Image was created using Partek Genomics Suite 7.19v (https://www.partek.com/partek-genomics-suite/).
