# Supplementary material for: Transcriptome and methylome analysis reveals three cellular origins of pituitary tumors
Source: Sci Rep. 2020 Nov 9;10:19373. doi: 10.1038/s41598-020-76555-8 (PMC7652879; doi:10.1038/s41598-020-76555-8)
Supplement: Supplementary file 3 — Supplementary information 3. [file 41598_2020_76555_MOESM3_ESM.pdf]

Transcriptome and methylome analysis reveals three cellular origins of pituitary tumors

Keiko Taniguchi-Ponciano[1], Sergio Andonegui-Elguera[1], Eduardo Peña-Martínez[1], Gloria Silva-Román[1], Sandra Vela-Patiño[1], Erick Gomez-Apo[2], Laura Chavez-Macias[2,3], Guadalupe Vargas-Ortega[4], Laura Espinosa-de-los-Monteros[4], Baldomero Gonzales-Virla[4], Carolina Perez[4], Aldo Ferreira-Hermosillo[1,4], Etual Espinosa-Cardenas[4], Claudia Ramirez-Renteria[1,4], Ernesto Sosa[4], Blas Lopez-Felix[5], Gerardo Guinto[5], Daniel Marrero-Rodríguez[1*] and Moises Mercado[1*]

1.- CONACyT-Unidad de Investigación Medica en Enfermedades Endocrinas, Hospital de Especialidades, Centro Medico Nacional Siglo XXI, Instituto Mexicano del Seguro Social.
2.- Área de Neuropatología, Servicio de Anatomía Patológica, Hospital General de México Dr. Eduardo Liceaga.
3.- Facultad de Medicina, Universidad Nacional Autonoma de México.
4.- Servicio de Endocrinologia, Hospital de Especialidades, Centro Medico Nacional Siglo XXI, Instituto Mexicano del Seguro Social.
5.- Servicio de Neurocirugia, Hospital de Especialidades, Centro Medico Nacional Siglo XXI, Instituto Mexicano del Seguro Social.

*Corresponding author
Daniel Marrero-Rodríguez, PhD.
CONACyT-Unidad de Investigación Médica en Enfermedades Endocrinas,
Hospital de Especialidades,
Centro Médico Nacional Siglo XXI
Instituto Mexicano del Seguro Social.
Av. Cuauhtémoc 330, Col. Doctores, México D.F. 06720
Phone: +54401021
e-mail: dan.mar57@gmail.com

*Corresponding author
Moisés Mercado, MD, FRCP(C)
Unidad de Investigación Médica en Enfermedades Endocrinas,
Hospital de Especialidades,
Centro Médico Nacional Siglo XXI
Instituto Mexicano del Seguro Social.
Av. Cuauhtémoc 330, Col. Doctores, México D.F. 06720
Phone: +54401021
e-mail: moises.mercado@endocrinologia.org.mx

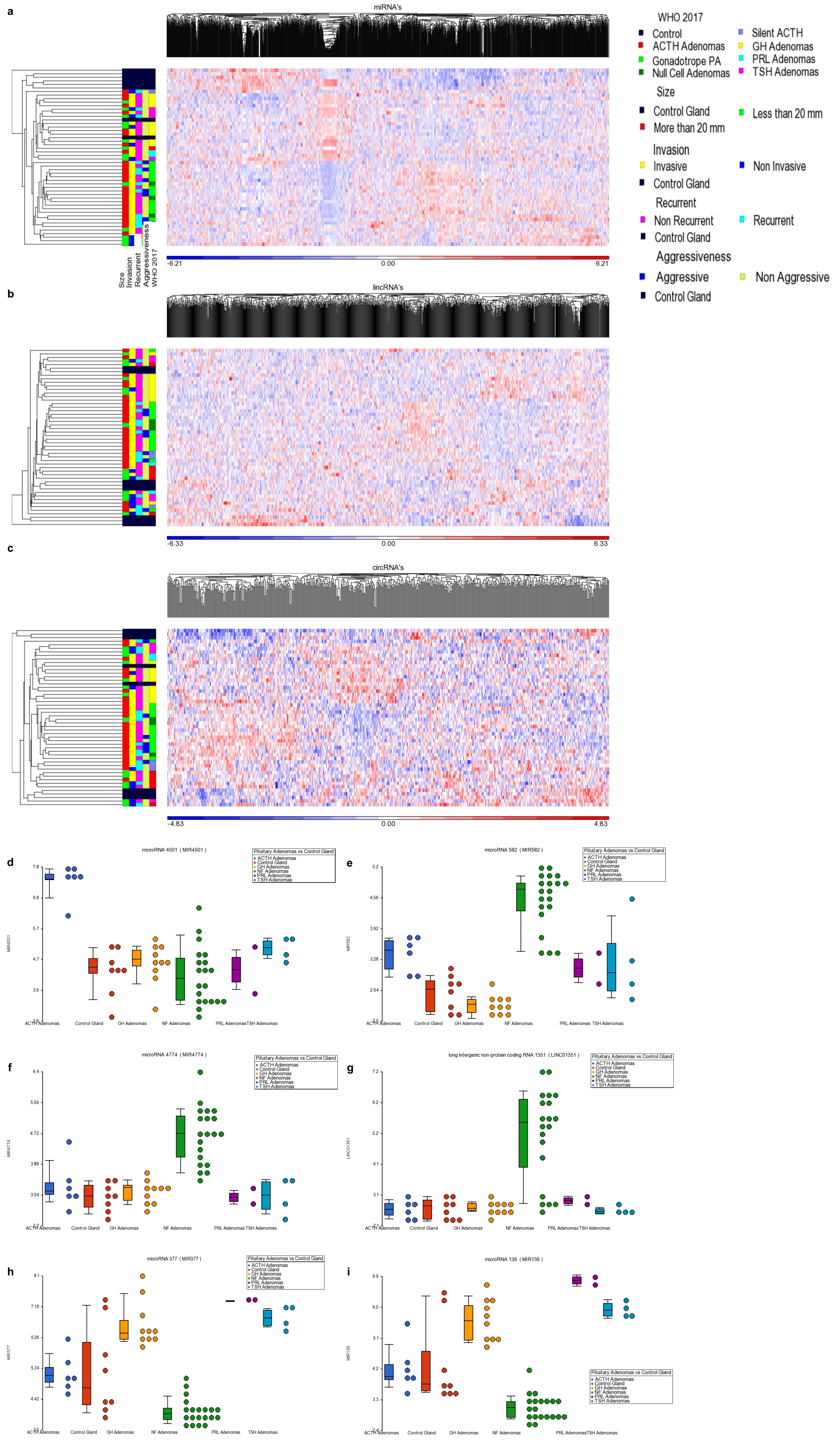

a
miRNA's
WHO 2017
Control
ACTH Adenomas
Gonadotrope PA
Null Cell Adenomas
Silent ACTH
GH Adenomas
PRL Adenomas
TSH Adenomas
Size
Control Gland
More than 20 mm
Less than 20 mm
Invasion
Invasive
Control Gland
Non Invasive
Recurrent
Non Recurrent
Control Gland
Recurrent
Aggressiveness
Aggressive
Control Gland
Non Aggressive
Size
Invasion
Recurrent
Aggressiveness
WHO 2017
-6.21
0.00
6.21
b
lincRNA's
-6.33
0.00
6.33
c
circRNA's
-4.83
0.00
4.83
d
microRNA 4501 (MIR4501)
Pituitary Adenomas vs Control Gland
ACTH Adenomas
Control Gland
GH Adenomas
NF Adenomas
PRL Adenomas
TSH Adenomas
MIR4501
e
microRNA 582 (MIR582)
MIR582
f
microRNA 4774 (MIR4774)
MIR4774
g
long intergenic non-protein coding RNA 1351 (LINC01351)
LINC01351
h
microRNA 377 (MIR377)
MIR377
i
microRNA 136 (MIR136)
MIR136
