# Supplementary material for: Transcriptome and methylome analysis reveals three cellular origins of pituitary tumors
Source: Sci Rep. 2020 Nov 9;10:19373. doi: 10.1038/s41598-020-76555-8 (PMC7652879; doi:10.1038/s41598-020-76555-8)
Supplement: Supplementary file 5 — Supplementary information 5. [file 41598_2020_76555_MOESM5_ESM.pdf]

Transcriptome and methylome analysis reveals three cellular origins of pituitary tumors

Keiko Taniguchi-Ponciano[1], Sergio Andonegui-Elguera[1], Eduardo Peña-Martínez[1], Gloria Silva-Román[1], Sandra Vela-Patiño[1], Erick Gomez-Apo[2], Laura Chavez-Macias[2,3], Guadalupe Vargas-Ortega[4], Laura Espinosa-de-los-Monteros[4], Baldomero Gonzales-Virla[4], Carolina Perez[4], Aldo Ferreira-Hermosillo[1,4], Etual Espinosa-Cardenas[4], Claudia Ramirez-Renteria[1,4], Ernesto Sosa[4], Blas Lopez-Felix[5], Gerardo Guinto[5], Daniel Marrero-Rodríguez[1*] and Moises Mercado[1*]

1.- CONACyT-Unidad de Investigación Medica en Enfermedades Endocrinas, Hospital de Especialidades, Centro Medico Nacional Siglo XXI, Instituto Mexicano del Seguro Social.
2.- Área de Neuropatología, Servicio de Anatomía Patológica, Hospital General de México Dr. Eduardo Liceaga.
3.- Facultad de Medicina, Universidad Nacional Autonoma de México.
4.- Servicio de Endocrinologia, Hospital de Especialidades, Centro Medico Nacional Siglo XXI, Instituto Mexicano del Seguro Social.
5.- Servicio de Neurocirugia, Hospital de Especialidades, Centro Medico Nacional Siglo XXI, Instituto Mexicano del Seguro Social.

*Corresponding author
Daniel Marrero-Rodríguez, PhD.
CONACyT-Unidad de Investigación Médica en Enfermedades Endocrinas,
Hospital de Especialidades,
Centro Médico Nacional Siglo XXI
Instituto Mexicano del Seguro Social.
Av. Cuauhtémoc 330, Col. Doctores, México D.F. 06720
Phone: +54401021
e-mail: dan.mar57@gmail.com

*Corresponding author
Moisés Mercado, MD, FRCP(C)
Unidad de Investigación Médica en Enfermedades Endocrinas,
Hospital de Especialidades,
Centro Médico Nacional Siglo XXI
Instituto Mexicano del Seguro Social.
Av. Cuauhtémoc 330, Col. Doctores, México D.F. 06720
Phone: +54401021
e-mail: moises.mercado@endocrinologia.org.mx

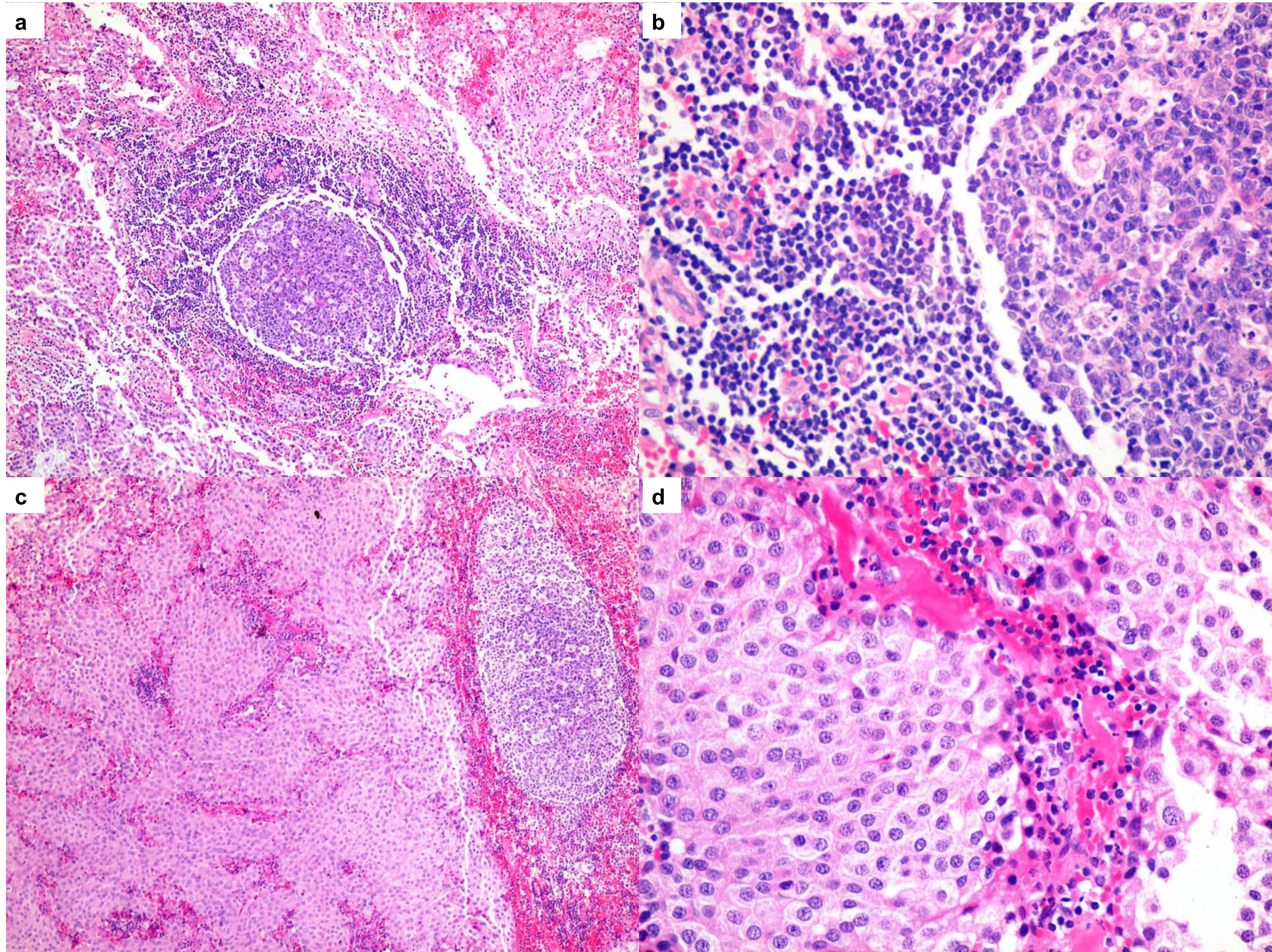
a
b
c
d
